# Clinical study of 2230 distal finger replants

**DOI:** 10.1186/1753-6561-9-S3-A61

**Published:** 2015-05-19

**Authors:** Wang Xin

**Affiliations:** 1 Department of Hand Surgery, Ningbo 6th Hospital, Ningbo, China

## 

Replantation is an ideal technique for reconstruction following fingertip amputation as it provides 'like for like' total reconstruction of the nail complex, bone pulp tissue and skin with no donor-site morbidity. This study is a clinical research on distal finger replantation and its classification, the purpose is to get better understanding of the neurovascular anatomy of distal finger, guide the clinical work, and improve the survival rate of replantation. Thirty-six damaged fingers with intact distal parts dissections were studied, focusing attention on the classification of the distal finger replantation. We summarized that the distal finger replantation can be classified into three types, based on the level at which it was amputated. Type I, level between the crease of DIPJ and the nail root. Type II, level between the nail root and the fingerprint center. Type III, level distal to the fingerprint center. From Jan 1995 to Mar 2013, 2230 cases (2645 fingers) of distal finger replantations were performed in our hospital, including 1434 fingers of type I, 737 fingers of type II, 374 fingers of type III. The artery diameter of type I and II refers to 0.3 mm - 0.6 mm, the dorsal and volar vein diameter refers to 0.3 mm - 0.8 mm. The artery of type III is showing dendritic, the diameter refers to 0.1 mm - 0.3 mm, vein diameter refers to 0.15 mm - 0.3 mm. The surgery includes debriding the wound, locating and tagging the vessels and nerves, shortening and fixing the bone, anastomosing the arteries, repairing the nerves, anastomosing the veins, and closing the wound. In the replantation of type I amputations, we could always find 1-2 arteries, 2 or more veins and 2 digital nerves which should be anastomosed. In type II amputations, 1-2 arteries, 1-2 veins and 2 digital nerves could be anastomosed, and sometimes we used veno-arteriolization technique. In type III amputations, the artery was really tiny, and we usually could only find 1 artery, 1 vein and 2 nerves which should be anastomosed. And also, if the distal vein was difficult to find, we anastomosed the distal artery to proximal vein. The postoperative treatment was much the same with the other replantations with a simiilar rehabilitation procedure. The replantation survival rate of the three types was 97.7%, 95% and 97% respectively. Follow-up period was 9-24 months. All the surviving replanted fingers achieved good sensory recovery and a satisfactory appearance. Conclusion: The replantation of the distal finger needs skilled microsurgical techniques. The Classification is important to choose the technique of replantation. Different types of fingertip amputation need different techniques. The vascular anastomosis is performed under the surgical microscope at 10x magnification in type I and II, and 16x magnification in type III. The venous drainage is usually a key point of the replantation. More veins should be anastomosed if possible. Veno-arteriolization technique is an effective way if we can only find the proximal vein. If it is really difficult to find any vein in type III, a tiny incision at the replanted fingertip that is kept bleeding after surgery can help drainage.

